# Effects of an Outpatient Diabetes Self-Management Education on Patients with Type 2 Diabetes in China: A Randomized Controlled Trial

**DOI:** 10.1155/2019/1073131

**Published:** 2019-01-17

**Authors:** Fan Zheng, Suixin Liu, Yuan Liu, Lihua Deng

**Affiliations:** Cardiac Rehabilitation Center of Rehabilitation Department, Xiangya Hospital at Central South University, Changsha, China

## Abstract

**Objective:**

This study is aimed at assessing the effectiveness of a simple outpatient diabetes self-management education programme.

**Methods:**

In the study, 60 patients with type 2 diabetes mellitus were randomly allocated into the control group (*n* = 30) and intervention group (*n* = 30). Regular and 2-session health education programmes were provided. The summary of diabetes self-care activity measure, problem areas in the diabetes scale, fasting blood glucose, postprandial 2 h blood glucose, and HbA1c were measured before and after the intervention to assess the effects of this 2-session diabetes education programme.

**Results:**

The total mean score of the summary of diabetes self-care activities measure was 17.60 ± 6.63 points. The problem areas in the diabetes scale revealed that the total mean score was 29.82 ± 15.22 points; 27% of the patients had diabetes-related distress, while 9% suffered from severe emotional distress. Compared with the control group, scores of the summary of diabetes self-care activities measure and problem areas in the diabetes scale, fasting blood glucose, postprandial 2 h blood glucose, and HbA1c were significantly improved in the intervention group after the intervention (*P* < 0.01).

**Conclusion:**

This study showed that the 2-session diabetes education programme could effectively improve the level of self-reported self-management, psychological distress, and glycemic control in patients with type 2 diabetes mellitus.

## 1. Introductions

Diabetes mellitus (DM) is the world's third-largest, chronic, noninfectious disease after cardiovascular diseases and cancer. DM and its complications threaten individual health and bring a heavy financial burden to the families and society [1–3]. Among the diabetic complications, the psychological disorders are usually neglected by the patients and their families [4, 5]. The latest Chinese study of 112 patients with type 2 diabetes mellitus (T2DM) showed that a general representation of anxiety, depression, and other psychological conditions existed among this population [6]. How to effectively improve the self-management behavior and negative mood of T2DM patients is the key goal of clinic work in China.

Diabetes self-management is considered as the cornerstone of T2DM management [7]. In reviewing the literature, the effectiveness of the didactic diabetes self-management education programmes was seemed to be rather weak when approaching T2DM and its related complications. The educational interventions that involved patient collaboration were more effective than didactic interventions in improving glycemic control [8–13]. Furthermore, a group-based diabetes self-management education programme was showed to produce improvements on clinical, lifestyle, and psychosocial outcomes in patients with T2DM [14, 15]. The researches in China also revealed that the family- and community-based diabetes self-management interventions were effective to improve the diabetes self-care behaviors in Chinese adults with type 2 diabetes [16, 17]. Indeed, the results showed that when self-management education was provided, age, education, knowledge, self-efficacy, economic factor, and social support should be considered to offer more appropriate intervention and to improve patients' behaviors in China [18, 19]. It is, consequently, suggested to develop a more comprehensive and participatory education programme and concentrate on some significant variables, i.e., self-management behavior and psychological performances [12, 13, 20].

The purpose of this study was to develop an interactive and participatory educational programme and evaluate the effects of this programme. An outpatient diabetes self-management education was subsequently conducted to guide these subjects in an appropriate, targeted, self-management manner and to improve the self-management level.

## 2. Methods

### 2.1. Participants

A single-blinded randomized controlled study, followed by CONSORT 2010 criteria [21], was approved by the Human Ethics Committee of Xiangya Hospital at Central South University. This study was conducted at the Cardiovascular Rehabilitation Clinic, Endocrinology Clinic, and Geriatrics Clinic at Xiangya Hospital, Central South University, from 2015 to 2017. The inclusion criteria were as follows: (I) All patients with T2DM should meet the T2DM diagnostic criteria established by the American Diabetes Association in 2010 [22]: (1) HbA1c ≥ 6.5%; (2) fasting blood glucose (FBG) ≥ 7.0 mmol/L; (3) postprandial blood glucose (PBG) ≥ 11.1 mmol/L in the oral glucose tolerance test (OGTT); and (4) typical hyperglycemia symptoms, such as polydipsia, polyuria, polyphagia, obvious weight loss, and random plasma glucose (RPG) ≥ 11.1 mmol/L; if the symptoms were not evident, criteria (1)–(3) should be repeatedly measured. (II) The patients were conscious, with complete behavior and cognitive ability. (III) The patients were willing to take the follow-up tests after three months. The exclusion criteria were as follows: (1) the presence of mental disorders; (2) complications of acute diabetes or inability to take care of themselves; (3) other serious diseases, such as severe cardiovascular and cerebrovascular diseases, severe kidney disease, cancer, and visual impairment due to complications of T2DM; (4) gestational diabetes; and (5) already received systematic diabetes education.

**Figure 1:**
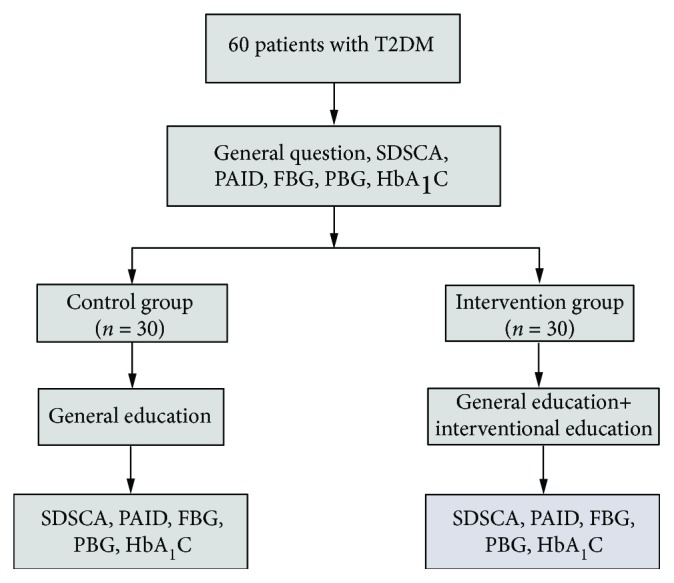
Presentation of the research flow chart.

Seventy-two potential participants were identified by the clinic physician for the study. However, 12 patients declined to take part in this study because of lack of time or inconvenient transportation. Finally, a total of 60 patients with T2DM were recruited in this study, and informed written consents were obtained from all these participants. A computer-generated randomization list with permuted, variable-size blocks was used to randomize the two groups. The allocation ratio of assignments was 1 : 1. The randomization and allocation concealment were performed by the statistician, and a therapist was in charge of enrollment and assignment of subjects to interventions.  Figure 1 shows the research flow.

### 2.2. Two-Group Experimental Design

Two groups, a preexperimental design and a postexperimental design, were used in this study. 60 patients with T2DM were randomly allocated into 2 groups: the control group (*n* = 30) and intervention group (*n* = 30). A questionnaire was distributed to the patients, and all of them were returned. This questionnaire included the general information such as gender, age, and educational background. Regular and interventional education programmes were applied to the control and intervention groups, respectively.

Regular education was given to the control group during their first clinic visit; this included general knowledge on diabetes disease process and treatment options; blood glucose monitoring; healthy lifestyle; preventing, detecting, and treating diabetes complications; and developing personalized strategies for decision-making [23]. The intervention group was given 2-session diabetes self-management education besides the regular education programme. Interventional education programme consisted of two types of courses: theory and practical. For the theory course, there were two timings for classes: the first class was given during the first clinic visit same as the control group, while the second class was given during the second clinic visit after 2 days. Each class lasted for 45 minutes. The second class consisted of group studies using an impressive PowerPoint-incorporated images and videos, presenting an overview knowledge of diabetes and additional details about self-management strategies, such as diet guidance, exercise guidance, and knowledge of hypoglycemia treatment, foot care, medication, and the blood glucose monitoring.

In the practical course, the educational tools were also culturally designed to be more appropriate and relevant for patients, including vivid models and individual practice. This course contains mainly two parts, one-on-one nutrition guidance and individualized exercise guidance. The one-on-one nutrition guidance was applied by the dietitians after the first theory class and lasted for 40 minutes. This guidance, developed for the Chinese diabetes population based on the American Diabetes Association [24], includes the definition and application of an exchangeable food portion in the diabetes diet planning assisted with the presentation of food simulation models. One piece of the “exchangeable food portion” is defined as “one serving for every 90 kcal of food produced.” The same type of the exchangeable food portion can be exchanged; the nutritional value is almost the same. The individual total energy required per day is calculated according to the individual weight and daily physical activity. Calculate the total individual servings of exchangeable food portion required per day = total calories per day divided by 90 (kcal/serving). Distribute the three nutrients in each serving according to the total calories: 50%–60% for carbohydrates, 15%–20% for proteins, and 20%–25% for fat. The daily serving is appropriately distributed into three to six meals, and every meal is supplied on time and based on the ration. Each food simulation model is marked with weight and calories, which makes the nutrition guidance more clear and vivid.

Individualized exercise guidance [25] includes making a personalized exercise prescription for each patient, according to the outcomes of cardiopulmonary exercise testing and the Borg scale of perceived exertion (Borg scale). Generally, the exercise training is performed three times a week and 60–90 min each time. Each training programme includes (1) warm-up session: low-intensity aerobic exercise for 5–10 min; (2) exercise session: aerobic exercise for 30 min at a moderate intensity, i.e., 50%–60% of maximal oxygen uptake (VO_2max_) and at a rate of 13–14 (somewhat hard of the Borg scale), and resistance exercise for 30 min at a moderate intensity, i.e., 50%–60% of 1RM; and (3) relaxation session for 10 min. The intervention group experienced a complete exercise training programme at the Cardiac Rehabilitation Center of Xiangya Hospital under therapist guidance and electrocardiogram monitoring. This individualized exercise guidance was applied after the second theory class and lasted for 60 minutes. Some instructions were mandatory: avoiding exercise on an empty stomach; knowing the identification and treatment of hypoglycemic episodes; and knowing how to adjust the insulin dose according to the exercise.

### 2.3. Outcomes

The SDSCA, PAIDs, FBG, postprandial 2 h blood glucose, and HbA1c tests were all performed to evaluate the effects of interventions for both groups before and after 3 months. The SDSCA scale [26] included five items: dietary behavior, exercise, medication adherence, blood glucose monitoring, and foot care. The Likert 7 rating scale was used, with scores ranging from 0 to 7, indicating “Don't do it at all” to “Complete it all.” The ranking method was as follows: total points > 28 scores (single item > 5.6 scores) were good, 21–28 was normal, and <21 (single item < 4.2 scores) was poor. Cronbach's *α* coefficients and the test-retest reliability coefficient of the SDSCA were 0.913 and 0.774 (*P* < 0.01), respectively [27].

PAID [28] was a self-administered, 20-item scale, and every item was scored from 0 to 4: 0 = no problem at all, 1 = a little problem, 2 = moderate problem, 3 = serious problem, and 4 = severe problem. The total score was the sum of all item scores multiplied by 1.25, with a range of 0–100 points. The higher the score, the more serious the associated psychological distress. Scores from 0 to 33 were believed to be normal, >33 meant that DM was accompanied by a related mental pain, and >44 indicated that the mental problems were quite severe. Cronbach's *α* coefficients and the test-retest reliability coefficient of PAID were 0.94 and 0.65 (*P* < 0.01), respectively [29].

After fasting for 12 hours, blood samples were obtained from cubital veins of the patients in the morning. HbA1C was measured by the low-performance liquid chromatography (DiaSTAT Hemoglobin A1C analyzer, Bio-Rad Laboratories Inc., Philadelphia, USA). FBG concentration of the serum was determined by the hexokinase method (BS300 biochemistry analyzer, Mindray Inc., Shenzhen, China). After 2-hour oral administration of 75 g of anhydrous glucose, a blood sample was taken from the cubital vein again to measure the postprandial 2 h blood glucose concentration (BS300 biochemistry analyzer, Mindray Inc., Shenzhen, China).

### 2.4. Descriptive Statistics

Qualitative information related to the research sample was presented as frequency and percentage. The quantitative data were described as the mean ± standard deviation (SD). SPSS statistical software, version 17.0 Windows (SPSS Inc., Chicago, IL, USA), was used for statistical analysis. The *t*/*χ*^2^ tests were used to compare the differences between the demographics and characteristics of two groups. *P* < 0.05 indicated that the difference was statistically significant.

## 3. Results

### 3.1. Baseline Information

The general information gained in this sample was as follows: the percentage of male was 55%, the average age was 52.22 ± 11.32 years, the systolic blood pressure was 134.09 ± 18.17 mmHg, the diastolic blood pressure was 84.43 ± 11.61 mmHg, and the body mass index was 26.33 ± 3.47 kg/m^2^. Most of the patients were rural residents (57%), nonsmokers (94%), physically inactive (87%), with a poorly controlled glycosylated hemoglobin level (53%), poor glucose control (52%), and a short disease course of no more than 5 years (52%) (Table 1).

The current treatments for T2DM were summarized as follows: restriction of total calorie intake combined with exercise training, oral antidiabetic agents (OADs), and insulin injection. Twelve percent of the sample undertook the restriction of total calorie intake combined with exercise training. Seventy-three percent of the sample took only OADs, which includes metformin, sulfonylureas, alpha-glucosidase inhibitors, thiazolidinediones, and meglitinides. They were used as monotherapy or in combinations to arrive at the best individualized prescription to achieve treatment goals. Last, fifteen percent of the sample took insulin injection with/without OADs (Table 1).

### 3.2. Self-Management Behavior and Mental Health of Patients

The present study showed that the average score of SDSCA was 17.60, and the overall score was low (<21 points is poor). The average score of PAID was 29.82 points (0~33 points are considered as normal), suggesting that the overall psychological condition of the sample was good. A further stratified analysis of the overall scores revealed that 27% of the sample had diabetes-related pain and 9% of them were severely emotionally disturbed (Table 2). These severely emotionally disturbed diabetics were all treated with insulin injection with/without OADs.

### 3.3. Effects of Outpatient Diabetes Self-Management Education Programme

The scores of SDSCA and PAID and blood glucose levels of the two groups were compared before and after 3 months to evaluate the effect. It was revealed that the scores of SDSCA and PAID, FBG, postprandial 2-h blood glucose, and HbA1c in the intervention group were all significantly (*P* < 0.01) improved after the intervention, as compared with those in the control group (Table 3).

## 4. Discussion

The first finding of this study was that the overall self-management behavior score of the sample was rather low, indicating poor self-management behavior of these patients. Compared with a previous study [30], the score in this study was even lower (17.60 ± 6.63 points vs. 22.0 ± 4.1 points in SDSCA), which might be related to the rural population with poor education, backward economy, and an undeveloped medical health system in the study sample. The second finding of this study was that these patients performed poorly on the psychological status, showing that 27% of the patients had negative emotions, of which 9% had severe diabetes distress. This was in line with previous studies, indicating that the incidence of anxiety disorder was 14% [31] in the diabetic population, the risk of depression in T2DM was 24% more than in nondiabetics [32], and more than 33% of T2DM had depressive symptoms [33]. In fact, when those severely emotionally disturbed diabetics were interviewed, they generally responded that they had the “fear of injection.” These patients were treated with insulin and reported the “injection-related uncomfortable feelings,” such as anxiety, scarring, sensitivity, pain, and bruising. Injection was perceived as restricting and interfering with their lives. They felt embarrassed about injecting in public, particularly when injecting with meals. They were worried about the inconvenience when travelling and the increased dependence on others especially when getting older. Based on the above fears of injection, the patients would rather take more OADs than inject insulin. Thus, they showed a poor adherence to injection compared to OADs. These negative emotions, to some degree, may have affected the patient's behavior of glycemic control, aggravated disease prognosis, and increased the patient's mental burden, forming a “vicious circle” [34].

Diabetes is a life-long disease. As the disease progresses, acute, chronic, and serious complications develop, which greatly weaken patients' confidence in the recovery. Patients gradually slack off in self-management behavior and reduce therapy compliance, which eventually leads to aggravation of the disease. Evidence supported the effectiveness of diabetes education and self-management programme on the self-management of T2DM, particularly in the short term. Thereinto, lifestyle advice on diet and exercise was at the core of first line. Because of the Chinese traditional view of dependence on the treatment with medication, diabetes self-management education in China is still not widely accepted and valued by the T2DM patients and their families. The current format of diabetes education in China focuses on a more didactic approach to educate patients about prescriptions and T2DM-related complications. This makes the diabetes self-management education even somehow difficult to execute and sustain by these patients. As showed in the present study, most of the patients were with lower education; thus, the diabetes self-management education should be easier to understand and follow in various forms. What is more, the cooperation between patients and providers should also be strengthened. It showed in the study that the educators could help patients relieve the fears of insulin injection and improve the injection technique to reduce physical discomfort, resulting in a better adherence to treatment and improved performance of self-management behaviors. Thus, patients should be encouraged to discuss their own problems, such as injection-related concerns, and providers should offer patients information about tools to reduce injection-related worries, preferably by directly showing to them. Patients and providers should work together to find the solutions.

On the other hand, most diabetes education studies in China are aimed at inpatients and the community population currently, with common patterns of collective instruction or individual guidance. These diabetes self-management education interventions usually involved a number of sessions over a long period and cost a lot of time and energy for the organizer. Hence, they were difficult to be promoted across the country. Patients were also reluctant to follow a long-term programme because of the transportation and the uncovered medical insurance; consequently, the rates of participation and compliance were relatively low [35]. There is currently not a unanimous diabetes self-management education developed by voluntary organizations or community groups [8]. Based on the aforementioned considerations, we employed one-to-one dietary guidance and individualized exercise instruction in the short-term outpatient education programme and investigated the effects on self-management behavior, psychological status, and glycemic control.

Dietary guidance used the “exchangeable food portion” method presented by the simulation food model. A recent study reported [36] that this type of dietary guidance applied more practical experiences and specific skills compared with the traditional Chinese education pattern. Consequently, it was easier for patients to understand and accept, resulting in improved glycemic control and lipid metabolism. This finding is in line with the results of a previous study, where the patients could make their own food services per day according to personal preferences after dietary guidance, shifting the boring and difficult-to-understand theory of dietary theories to concrete and easy-to-use individual practice. In the exercise section, a combined exercise training programme was applied, i.e., moderate intensity aerobic exercise combined with resistance exercise in the present study. Under the guidance of medical staff and continuous ECG monitoring, the patients in the intervention group took a full set of exercise training programme. This type of exercise guidance concentrated on individualized exercise education and guidance.

For the sake of economic issue, the intervention programme in the present study took place only over two turns along with the patient's first visit and return visit to the outpatient department. This intervention programme integrated the advantages of the two education patterns, combining collective instructions with individual guidance (individualized diet guidance and exercise practice). Although taking only two sessions, the effects of the outcomes were significant, i.e., higher participation and better compliance by the patients, improved self-management behaviors and psychological conditions, and lower blood glucose levels. The spotlight of this diabetes self-management education programme was that it approached patients' own risk factors and encouraged and educated patients to act on the risk factors of most importance to them, i.e., lifestyle, diet, and physical activity.

The strength of this study was that it provided new ideas and practical experience for diabetes health education in clinic work. As for some undeveloped districts or countries, in where patients have poor education and are under poor economic status, it is important to encapsulate a patient-centered approach to diabetes self-management to maximize the profits of these patients and greatly improve the participation and adherence to diabetes self-management education. However, there were still some limitations in this study, primarily related to the composition of the analyzed sample, that is, regionalism, resources of the patients (limited to outpatients), and so on. Additionally, the sample of this study is small. Due to the limited sample in this study, we did not compare the self-management behaviors, psychological condition, glucose control, and the effects of educational intervention in different types of antidiabetic therapy (e.g., insulin injection vs. OADs). In this sense, even the patients treated with insulin seemed to experience higher levels of emotional distress in the study, but unfortunately, we could not make such a conclusion because of the limited samples and lack of statistical analysis. Last, the intervention patterns in this study were limited, and the observation period was relatively short. Future studies will use some intelligent medical devices to track the vital signs and intervention effects of a large sample of T2DM patients for a longer time.

In conclusion, the overall level of self-management of patients with T2DM is still relatively low. Some patients have obvious negative emotions. The short-term diabetes self-management education for outpatients can effectively improve the level of self-management, psychological condition, and glycemic control in T2DM.

## Figures and Tables

**Table 1:** General information about the sample (*n* = 60).

Item	CG (*n* = 30)	IG (*n* = 30)	*t*/*χ*^2^	*P*
Age (years)	51.92 ± 12.30	52.52 ± 10.46	0.20	*P* > 0.05
Disease course (years)	2.33 ± 1.58	2.59 ± 1.89	0.58	*P* > 0.05
Weight (kg)	72.46 ± 1.96	71.56 ± 1.64	-1.93	*P* > 0.05
Male : female	17 : 13	16 : 14	0.07	*P* > 0.05
Education (years)	9.52 ± 4.20	9.38 ± 3.70	-0.14	*P* > 0.05
Urban : rural	11 : 19	15 : 15	1.09	*P* > 0.05
Physically active : inactive	5 : 25	3 : 27	0.58	*P* > 0.05
Smoker : nonsmoker	1 : 29	3 : 27	1.07	*P* > 0.05
Insulin injection : OADs	4 : 21	5 : 23	0.03	*P* > 0.05

CG: control group; IG: intervention group; OADs: oral antidiabetic agents.

**Table 2:** Results of diabetes self-care activities measure and problem areas in diabetes (*n* = 60).

Item	Rating scale (points)	Mean ± SD (points)	Stratification percentage		
			Good (normal, %)	Fair (pain, %)	Poor (severe, %)
Dietary control	0–7	4.18 ± 1.73	28.5	17.0	54.5
Physical activity	0–7	3.18 ± 2.35	25	9	66.0
Medication adherence	0–7	5.25 ± 1.66	79	2	19
Blood glucose monitoring	0–7	2.44 ± 1.26	19	4	77
Foot care	0–7	2.40 ± 1.02	15	4	81
SDSCA score	0–35	17.60 ± 6.63	8	28	64
PAID score	0–100	29.82 ± 15.22	73	27	9

The ranking method: summary of diabetes self-care activities measure scores: total points > 28 or single item > 5.6 scores was good, 21–28 was normal, and <21 or single item < 4.2 was poor. Problem areas in diabetes scores between 0 and 33 were believed to be normal, >33 points meant a diabetes-related mental pain, and >44 points indicated severe mental problems.

**Table 3:** SDSCA score, PAID score, and blood glucose level of the two groups before and after the intervention (mean ± SD, *n* = 60).

Item	Before		*t*	*P*	After		*t*	*P*
	CG (*n* = 30)	IG (*n* = 30)			CG (*n* = 30)	IG (*n* = 30)		
SDSCA (scores)	17.72 ± 7.21	17.47 ± 6.11	-0.14	*P* > 0.05	18.62 ± 1.31	22.80 ± 4.86	4.55	*P* < 0.01
Dietary control (scores)	4.13 ± 1.86	4.23 ± 1.62	0.22	*P* > 0.05	4.53 ± 0.86	5.75 ± 0.28	7.39	*P* < 0.01
Physical activity (scores)	3.25 ± 2.47	3.11 ± 2.27	-0.23	*P* > 0.05	3.82 ± 1.27	5.37 ± 0.56	6.12	*P* < 0.01
Medication adherence (scores)	5.25 ± 2.03	5.24 ± 1.21	-0.02	*P* > 0.05	5.75 ± 2.33	6.52 ± 0.81	1.71	*P* > 0.05
Blood glucose monitoring (scores)	2.55 ± 1.41	2.33 ± 1.12	-0.67	*P* > 0.05	2.65 ± 0.55	2.55 ± 1.08	-0.45	*P* > 0.05
Foot care (scores)	2.52 ± 1.03	2.28 ± 1.01	-0.91	*P* > 0.05	2.63 ± 1.02	3.43 ± 0.85	3.30	*P* < 0.01
PAID (scores)	30.72 ± 15.86	28.91 ± 14.76	-0.46	*P* > 0.05	26.57 ± 12.50	21.15 ± 0.25	-2.37	*P* < 0.05
FBG (mmol/L)	8.48 ± 1.10	8.35 ± 1.21	-0.44	*P* > 0.05	7.48 ± 1.10	6.11 ± 0.72	-5.72	*P* < 0.01
PBG (mmol/L)	13.96 ± 3.72	13.67 ± 1.12	-0.41	*P* > 0.05	12.16 ± 1.72	9.04 ± 1.40	-7.71	*P* < 0.01
HbA1c (mmol/L)	8.48 ± 0.40	8.30 ± 1.02	-0.89	*P* > 0.05	8.53 ± 0.72	6.34 ± 0.87	-10.62	*P* < 0.01

CG: control group; IG: intervention group; FBG: fasting blood glucose; PBG: postprandial blood glucose; HbA1c: glycosylated hemoglobin. *P* < 0.05 was considered statistically significant.

## Data Availability

The data used to support the findings of this study are included within the article.
